# Neurocognitive function in HIV-infected persons with asymptomatic cryptococcal antigenemia: a comparison of three prospective cohorts

**DOI:** 10.1186/s12883-017-0878-2

**Published:** 2017-06-12

**Authors:** Martha P. Montgomery, Noeline Nakasujja, Bozena M. Morawski, Radha Rajasingham, Joshua Rhein, Elizabeth Nalintya, Darlisha A. Williams, Kathy Huppler Hullsiek, Agnes Kiragga, Melissa A. Rolfes, Renee Donahue Carlson, Nathan C. Bahr, Kate E. Birkenkamp, Yukari C. Manabe, Paul R. Bohjanen, Jonathan E. Kaplan, Andrew Kambugu, David B. Meya, David R. Boulware, on behalf of the COAT and ORCAS Trial Teams

**Affiliations:** 1 Division of Infectious Diseases and International Medicine, Department of Medicine, University of Minnesota, 3-222 MTRF, 2001 6th St SE, Minneapolis, MN 55455 USA; 2 Infectious Disease Institute, Makerere University, Kampala, Uganda; 3 Department of Medicine, School of Medicine, Makerere University College of Health Sciences, Kampala, Uganda; 4 Department of Psychiatry, School of Medicine, Makerere University College of Health Sciences, Kampala, Uganda; 5 School of Public Health, University of Minnesota, Minneapolis, MN USA; 6 Johns Hopkins School of Medicine, Baltimore, MD USA; 7 Division of Global HIV and TB, Center for Global Health, Centers for Disease Control and Prevention, Atlanta, GA USA; 8 Infectious Disease Institute, Mulago Hospital Complex, Kampala, Uganda

**Keywords:** AIDS dementia complex, Neurocognitive disorders, Cryptococcal meningitis, HIV, Neuropsychological tests, Cryptococcus

## Abstract

**Background:**

HIV-infected persons with detectable cryptococcal antigen (CrAg) in blood have increased morbidity and mortality compared with HIV-infected persons who are CrAg-negative. This study examined neurocognitive function among persons with asymptomatic cryptococcal antigenemia.

**Methods:**

Participants from three prospective HIV cohorts underwent neurocognitive testing at the time of antiretroviral therapy (ART) initiation. Cohorts included persons with cryptococcal meningitis (*N* = 90), asymptomatic CrAg + (*N* = 87), and HIV-infected persons without central nervous system infection (*N* = 125). Z-scores for each neurocognitive test were calculated relative to an HIV-negative Ugandan population with a composite quantitative neurocognitive performance Z-score (QNPZ-8) created from eight tested domains. Neurocognitive function was measured pre-ART for all three cohorts and additionally after 4 weeks of ART (and 6 weeks of pre-emptive fluconazole) treatment among asymptomatic CrAg + participants.

**Results:**

Cryptococcal meningitis and asymptomatic CrAg + participants had lower median CD4 counts (17 and 26 cells/μL, respectively) than the HIV-infected control cohort (233 cells/μL) as well as lower Karnofsky performance status (60 and 70 vs. 90, respectively). The composite QNPZ-8 for asymptomatic CrAg + (−1.80 Z-score) fell between the cryptococcal meningitis cohort (−2.22 Z-score, *P =* 0.02) and HIV-infected controls (−1.36, *P =* 0.003). After four weeks of ART and six weeks of fluconazole, the asymptomatic CrAg + cohort neurocognitive performance improved (−1.0 Z-score, *P <* 0.001).

**Conclusion:**

Significant deficits in neurocognitive function were identified in asymptomatic CrAg + persons with advanced HIV/AIDS even without signs or sequelae of meningitis. Neurocognitive function in this group improves over time after initiation of pre-emptive fluconazole treatment and ART, but short term adherence support may be necessary.

**Electronic supplementary material:**

The online version of this article (doi:10.1186/s12883-017-0878-2) contains supplementary material, which is available to authorized users.

## Background

Cryptococcal meningitis is one of the leading causes of death from HIV/AIDS in sub-Saharan Africa [1, 2] causing 15–20% of AIDS-related mortality [3]. In the United States, cryptococcal meningitis is more common than all causes of bacterial meningitis combined (1.1 vs. 0.73 per 100,000 people, respectively) [4, 5]. Yet cryptococcal disease is potentially preventable as cryptococcal antigen (CrAg) is detectable in the peripheral blood weeks to months prior to the onset of symptomatic meningitis. Although many of these CrAg + persons exhibit no clinical signs or symptoms despite the presence of early disseminated infection, CrAg + persons have increased risk of developing meningitis or death compared to HIV-infected persons without cryptococcal antigenemia [6–9].

The World Health Organization (WHO) and U.S. HIV guidelines recommend routine CrAg screening for HIV-infected persons with <100 CD4 T cells/μL who are not receiving effective antiretroviral therapy (ART), along with pre-emptive fluconazole therapy for those screening CrAg + [10, 11]. Despite these recommendations, many gaps remain in understanding the morbidity and clinical course of asymptomatic CrAg + persons.

Persons living with HIV, regardless of their CrAg serostatus, have increased risk of neurocognitive deficits, presumably through the direct toxic effect of the HIV virus itself and consequent inflammation. The wide array of observed disorders is referred to collectively as HIV-associated neurocognitive disorder (HAND), which ranges from mild cognitive impairment to the more extreme HIV-associated dementia [12]. One prevalence study estimated that 31% of people living with HIV in Uganda have HIV dementia and that people with older age and lower CD4 counts are at higher risk [13].

Opportunistic infections of the central nervous system (CNS) create potential for further damage, and HIV-infected persons with cryptococcal meningitis, specifically, are known to have increased neurocognitive deficits compared with their HIV-infected, CrAg-negative peers [14, 15]. It is not known whether cryptococcal antigenemia increases the risk of neurocognitive dysfunction in asymptomatic CrAg + persons, which may have an implication for additional needed support for general HIV care. The purpose of this study was to 1) investigate neurocognitive changes among HIV-infected persons with asymptomatic cryptococcal antigenemia, and 2) compare neurocognitive performance of these asymptomatic CrAg + persons to HIV-infected persons with cryptococcal meningitis and those without CNS infection.

## Methods

This study utilized three cohorts of HIV-infected ART-naïve persons enrolled in prospective clinical trials and cohort studies in Kampala, Uganda. All participants provided written, informed consent prior to enrollment. Studies were approved by institutional review boards in Uganda and Minnesota.

### Study populations

The first population consisted of HIV-infected persons hospitalized with cryptococcal meningitis who survived to the one-month follow-up visit. These participants were prospectively enrolled into a clinical trial to determine optimal timing of ART after the first episode of cryptococcal meningitis (ClinicalTrials.gov: NCT01075152) [14, 16]. Cryptococcal meningitis was diagnosed based on cerebrospinal fluid (CSF) culture and/or CSF CrAg lateral flow assay (Immy, Norman, Oklahoma). Participants diagnosed with cryptococcal meningitis were treated with combined amphotericin B deoxycholate (0.7–1.0 mg/kg/day) plus fluconazole 800 mg/day in a two-week induction therapy regimen, followed by enhanced consolidation therapy of fluconazole of 800 mg/day for ~3 weeks, then 400 mg/day for 8 weeks, and then secondary prophylaxis with fluconazole 200 mg/day. ART was initiated at either 1–2 weeks following cryptococcal meningitis diagnosis or 5 weeks after diagnosis, based on the randomized trial arm in the main clinical trial. Baseline neurocognitive testing was performed at approximately 1 month after meningitis diagnosis. We have previously shown that there was no difference in baseline or longitudinal neurocognitive function based on early vs. deferred ART. Additional study details have been published previously [14, 16].

The second study population included HIV-infected, ART-naïve persons who were CrAg + in plasma after lab-based reflexive screening among persons with CD4 < 100 cells/μL, but without symptoms of cryptococcal meningitis (ClinicalTrials.gov: NCT01535469). Participants were enrolled prospectively and followed for 6 months. All asymptomatic CrAg + persons received pre-emptive fluconazole therapy at 400 mg twice daily for 2 weeks, followed by 400 mg daily for the subsequent 8 weeks. ART was started 2 weeks after initiation of fluconazole. Neurocognitive testing was performed at the ART initiation visit and then again 4 weeks after ART initiation.

Third, to account for the potential effect of HAND, we included a population of HIV-infected persons enrolled in an outpatient clinic setting [17]. HIV-infected adults were screened consecutively for enrollment, and those with clinical signs or symptoms of meningitis or with a history of CNS infection were excluded. Additional exclusion criteria included active psychiatric disease, alcoholism, or physical deficits that would interfere with neurocognitive testing. In this cohort, neurocognitive testing was performed once prior to ART initiation.

### Neurocognitive testing

Participants in all three cohorts underwent outpatient neurocognitive testing with a neuropsychological battery of tests. Testing for all three cohorts and for the retesting of the asymptomatic CrAg + cohort was performed by the same team using the same study forms and was conducted in participant’s native language. The assessments explored the following neurocognitive domains: Attention span: Digit Span Forward and Backward [18] and Color Trails 1 [19]. Processing speed: Digit Symbol test [18] and Color Trails 1 [19]. Executive function: Color Trails 2 [19]. Verbal learning: WHO-UCLA (University of California, Los Angeles) Auditory Verbal Learning Test-Revised [20]. Language fluency: Semantic Verbal Fluency [21]. Motor speed: Finger Tapping [22]. Fine motor skills: Grooved Pegboard [23]. Additional file 1 provides a description of each of these tests. Each test score was compared with a standard reference population of HIV-negative Ugandans matched on age and education to produce an age and education adjusted Z-score [24, 25]. Negative Z-scores indicate a worse performance compared with the reference population. A composite score using all eight tests was calculated to create a Quantitative Neurological Performance Z-score (QNPZ-8 score) [26].

In addition to neurocognitive testing, all three populations were evaluated for functional status with the Karnofsky performance status scale [27]. Participants were screened for dementia using the International HIV Dementia Scale (IHDS) [24]. Total scores range from 0 to 12 with a score ≤ 10 being 80% sensitive and 55% specific for HIV dementia. Depression was assessed using the Center for Epidemiologic Studies Depression (CES-D) Scale [28], which ranges from 0 to 60. Higher scores correspond with increased chance of depression, and a score of 16 or greater has good sensitivity and specificity for identifying clinical depression [29, 30]. All enrolled participants who completed baseline neurocognitive testing were included in the analyses.

### Data analysis

Data were analyzed using STATA/IC version 9.2 (StataCorp, College Station, TX, USA) and are available online in a supplemental file (Additional file 2). Participants who initiated the neurocognitive tests but were too sick to complete all tests attempted as many neurocognitive tests as possible and skipped the remainder. For each skipped/uncompleted test, participants were assigned a value of −2 standard deviations below the cohort mean for that test, as has been done previously [14]. Comparisons of variables among populations were made using unpaired Student’s t-tests, Mann-Whitney U tests, and chi-square tests as appropriate. Linear regression was used to identify factors associated with QNPZ-8 score. Comparisons of neurocognitive performance over time were made using paired t-tests of Z-scores.

## Results

Participants with asymptomatic cryptococcemia were slightly younger in age (median 31 years, interquartile range (IQR): 27–39 years) compared with participants with cryptococcal meningitis (36 years, IQR: 30–40) and HIV-infected controls (37, IQR: 32–41) (Table 1). There were significantly more women in the HIV control population (68% women) relative to patients with *Cryptococcus* infection. Median education level ranged from 7 to 8 years in the three groups. CD4 count, Karnofsky score, CES-D scale, and IHDS were variable among the three groups. Participants with cryptococcal meningitis had the lowest median CD4 count (17 cells/μL, IQR: 7–79), the lowest median Karnofsky score (60, IQR: 50–70), the highest proportion of participants with IHDS < =10 (92%), and the highest median scores for depression (CES-D: 23, IQR: 16–30). At the other end of the spectrum, the HIV-infected controls had the lowest proportion with IHDS < =10 (66%), the lowest depression scale scores (CES-D: 9, IQR: 5–17), highest Karnofsky performance score (90, IQR: 90–90), and significantly higher median CD4 counts (233 cells/μL, IQR: 183–297) compared with the other two study populations. Overall, asymptomatic CrAg + participants fell in between the two populations and were notably more similar to cryptococcal meningitis survivors with regard to CD4 count (median 26 cells/μL, IQR: 9–59).

**Table 1:** Background and clinical characteristics of three study populations

	Cohort A			Cohort B			Cohort C				
	Cryptococcal meningitis (*N* = 90)			Asymptomatic CRAG+ (*N* = 87)			HIV+ controls (*N* = 125)				
	N	% or Median	(IQR)	N	% or Median	(IQR)	N	% or Median	(IQR)	A vs B *P*-value	B vs C *P*-value
Age (years)	90	36	(30–40)	87	31	(27–39)	125	37	(32–41)	0.02	<0.001
Sex	90			87			125				
Male		60%			51%			32%			
Female		40%			49%			68%		0.21	0.007
Education level (years)	90	8	(4–11)	87	7	(3–11)	125	8	(6–11)	0.15	0.05
< 7 years		36%			45%			27%			
7 to 12 years		51%			44%			62%			
> 12 years		13%			11%			10%		0.45	0.02
CD4 count (cells/uL)	79	17	(7–79)	86	26	(9–59)	125	233	(183–297)	0.93	<0.001
Karnofsky score	90	60	(50–70)	87	70	(60–80)	125	90	(90–90)	<0.001	<0.001
40		1%			0%			0%			
50		28%			11%			0%			
60		36%			20%			1%			
70		29%			26%			0%			
80		3%			29%			2%			
90		3%			14%			97%			
International HIV dementia scale	88			87			125				
< =10		92%			75%			66%			
> 10		8%			25%			34%		0.002	0.20
CES depression scale	88	23	(16–30)	87	18	(10–26)	125	9	(5–17)	0.007	<0.001
< 16		25%			41%			70%			
> =16		75%			59%			30%		0.02	<0.001

Twenty participants from the cryptococcal meningitis cohort and 28 asymptomatic CrAg + participants were excluded due to insufficient data to calculate a QNPZ-8 score. *P*-values were calculated from student’s T-test (comparing means), Mann-Whitney U test, and chi-square test as appropriate

**Fig. 1:**
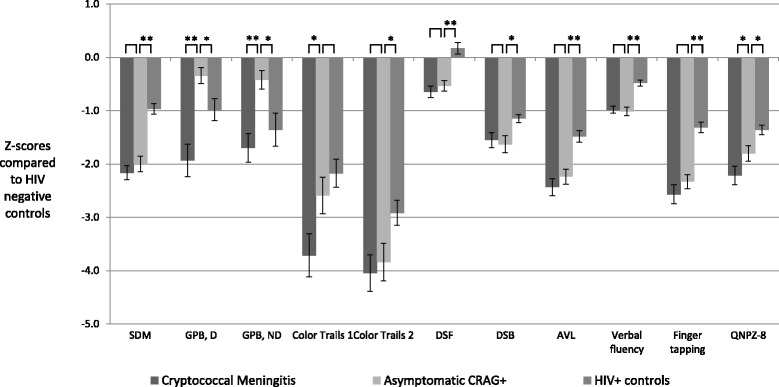
Neurocognitive test Z-scores according CrAg status. SDM – Symbol digit modalities. GPB, D – Grooved pegboard, dominant hand. GPB, ND – Grooved pegboard, non-dominant hand. DSF – Digit span forward. DSB – Digit span backward. AVL – Auditory visual learning. QNPZ-8 – Quantitative neurological performance Z-score on 8 modalities. Error bars indicate +/− one standard error. The number of imputed scores for each study ranged from 2 to 41 for CrAg + with meningitis, 1–34 for CrAg + asymptomatic, and 0–2 for HIV+ controls. Imputed scores for persons who started but could not complete a neurocognitive test were two standard deviations the group mean for the test. Numbers represent Z-scores referenced against age and education adjusted, HIV-negative Ugandan population norms. * < .05 ** < .0001

Individual pre-ART neurocognitive test Z-scores for each population are displayed in Fig. 1 (see Additional file 3). Asymptomatic CrAg + persons performed significantly worse than HIV-infected controls in every domain except for the Color Trails 1 and Grooved Pegboard tests. In addition, asymptomatic CrAg + persons did not significantly differ from cryptococcal meningitis survivors in any measure except for the Grooved Pegboard and Color Trails 1 tests (i.e., domains of speed of information processing, attention, fine motor). With respect to the composite QNPZ-8 score, the three study populations were significantly different from each other, with cryptococcal meningitis survivors performing the worst (QNPZ-8 = −2.22 vs −1.80 among asymptomatic CrAg+, *P =* 0.02) and HIV-infected controls performing the best (QNPZ-8 = −1.36, *P =* 0.003 compared with asymptomatic CrAg+).

There were slight demographic and other variable differences across the three cohort groups, thus a multiple linear regression analysis was performed to assess for independent risk factors associated with decreased neurocognitive performance (Table 2). In an adjusted multivariable model, the QNPZ-8 scores for HIV-infected controls remained significantly higher than QNPZ-8 scores for asymptomatic CrAg + persons (QNPZ-8: +0.42, 95% confidence interval (CI): 0.03 to 0.81). The asymptomatic CrAg + persons did not statistically differ from persons with recent cryptococcal meningitis (QNPZ-8: -0.26, 95% CI: -0.59 to 0.07). For each 10 year-increase in age, participants’ QNPZ-8 decreased by −0.31 (95% CI: -0.48 to −0.15), and participants with CES-D depression scores ≥16 had a mean difference of −0.56 QNPZ-8 score (95% CI: -0.82 to −0.30). In the multivariable model, CD4 count was not significantly associated with neurocognitive function after adjusting for study cohort (QNPZ-8 = 0.02 per 100 CD4 cells/μL, 95% CI: -0.10 to 0.14; *P =* 0.79). Baseline pre-ART plasma CrAg titer was also not associated with QNPZ-8 score among the CrAg + and cryptococcal meningitis cohorts (*P =* 0.41, data not shown).

**Table 2:** Simple and multiple linear regression analyses estimating the relationship of patient characteristics with composite neurocognitive function, QNPZ-8 score

Variable	N	Unadjusted univariate estimate^a^	95% confidence interval	*P* -value	Adjusted estimate^a^	95% confidence interval	*P* -value
Study Population	302						
Cryptococcal meningitis (*N* = 90)		−0.42	(−0.74, −0.09)	0.01	−0.26	(−0.59, 0.07)	0.12
Asymptomatic CRAG (*N* = 87)		Referent	- -		Referent	- -	
HIV positive controls (*N* = 125)		0.44	(0.14, 0.74)	0.004	0.42	(0.03, 0.81)	0.04
Age (per 10 years)	302	−0.20	(−0.37, −0.03)	0.02	−0.31	(−0.48, −0.15)	<0.001
Gender (Men as referent group)	302	0.01	(−0.25, 0.27)	0.94	−0.21	(−0.47, 0.04)	0.11
CD4 count (per 100 increase)	290	0.16	(0.07, 0.26)	0.001	0.02	(−0.10, 0.14)	0.79
Depression^b^	300	−0.77	(−1.02, −0.53)	<0.001	−0.56	(−0.82, −0.30)	<0.001

^a^Each estimate corresponds with the change in QNPZ-8 score for each incremental change in the variable

^b^Depression modeled as a binary variable comparing using CES-D score > = 16 versus <16 as referent

Lastly, we examined neurocognitive performance among asymptomatic CrAg + persons over time, comparing the pre-ART time point after 2 weeks of fluconazole and a time after 6 weeks of fluconazole plus 4 weeks of ART (Table 3). All measures showed significant improvement after 4 weeks of ART, except for Digit Span Forward, which tests the domains of working memory and attention. The overall QNPZ-8 score improved in the first 4 weeks of ART by +0.7 standard deviations of the population (*P <* 0.001). The baseline characteristics for asymptomatic CrAg + persons also improved significantly over 4 weeks. Average CD4 count increased from 32 cells/μL to 80 cells/μL (*P <* 0.001) and average depression scores decreased from 19 to 16 (*P =* 0.04). Mean Karnofsky performance scores modestly increased from 72 to 75 out of a 100-point scale (*P =* 0.04), indicating that participants on average were able to care for themselves but unable to carry on normal activity or to do active work.

**Table 3:** Population characteristics and average neurocognitive test Z-scores for 73 persons with asymptomatic cryptococcal antigenemia

	Pre-ART visit week 2 of fluconazole		Week 4 of HIV therapy week 6 of fluconazole		Intra-person change		
Population Characteristics	Average	SD	Average	SD	Change	SD	*P*-value
CD4 count (cells/uL), *N* = 60	32	±30	80	±74	47	±66	<0.001
Karnofsky score, *N* = 73	72	±12	75	±11	2.5	±10	0.04
CES depression scale, *N* = 73	19	±10	16	±10	−3.0	±12	0.04
Neurocognitive Tests							
Symbol digit modalities	−1.9	±1.2	−1.5	±1.5	0.4	±1.0	0.002
Grooved pegboard dominant	−0.3	±1.3	0.1	±1.1	0.4	±1.4	0.02
Grooved pegboard non-dominant	−0.4	±1.5	-0.02	±1.3	0.3	±1.3	0.03
Color Trails 1	−2.4	±2.6	−1.0	±1.7	1.4	±2.3	<0.001
Color Trails 2	−3.7	±2.6	−1.9	±1.6	1.7	±2.1	<0.001
Digit span forward	−0.5	±0.9	−0.3	±0.9	0.2	±0.9	0.15
Digit span backward	−1.4	±1.4	−1.1	±1.3	0.3	±1.2	0.03
Auditory verbal learning	−2.2	±1.4	−1.2	±1.5	1.0	±1.2	<0.001
Verbal fluency	−0.9	±0.7	−0.6	±0.7	0.3	±0.7	0.005
Finger tapping	−2.3	±1.1	−1.9	±1.0	0.4	±1.1	0.002
QNPZ-8	-1.7	±1.0	−1.0	±0.8	0.7	±0.7	<0.001

Numbers represent Z-scores referenced against age and education adjusted, HIV-negative Ugandan population norms. Initial testing was performed at the pre-ART visit which was 2 weeks after diagnosis of cryptococcal antigenemia and receipt of fluconazole pre-emptive therapy. Statistical testing by paired t-test

## Discussion

In this analysis of HIV-infected persons, we found that asymptomatic cryptococcal antigenemia is associated with more neurocognitive deficits compared with HIV-infected persons without a history of CNS infection. On several measures of neurocognitive function, HIV-infected CrAg + persons did not differ significantly from survivors of cryptococcal meningitis who were tested at 1 month after meningitis diagnosis [14]. Secondly, longitudinal assessment of asymptomatic CrAg + persons found that they had significant improvement in neurocognitive function on most neurocognitive measures after the first 4 weeks of ART. These findings have important practical implications. Persons with very advanced AIDS with asymptomatic cryptococcal antigenemia or recent history of cryptococcal meningitis have substantial neurocognitive dysfunction with severe deficits in executive function, verbal learning, and speed of information processing. With their neurocognitive dysfunction, this group of cryptococcal survivors and asymptomatic CrAg + persons likely require additional support to initially manage their ART and HIV care in order to avoid poor ART outcomes.

There are several potential explanations for the observation that asymptomatic CrAg + persons perform worse than HIV-infected persons without cryptococcal infection. Initial exposure to *Cryptococcus* is through the respiratory system, and the development of antigenemia results from dissemination from the respiratory system to the blood stream [31]. The course of disease seen thereafter is variable in time course, and asymptomatic CrAg + persons likely form a spectrum of infection – from those without any CNS penetration of *Cryptococcus* to persons with subclinical cryptococcal infection of the brain and meninges. More advanced HIV states are likely to result in increased CSF inflammation, which may increase risk of neurocognitive impairment or HAND [12]. Thus, the decreased cognitive function in asymptomatic CrAg + persons could be explained, at least in part, by increased prevalence of HAND.

One could similarly postulate that asymptomatic CrAg + persons perform poorly on neurocognitive testing because they have more advanced HIV with lower CD4 counts and functional performance. After adjusting for CD4 count, the CrAg + cohort effect remained while the effect of CD4 disappeared. This is most likely the result of “aggregation bias” wherein the cohort is too intricately related to the CD4 count to make a valid comparison. Unfortunately, there was an insufficient number of participants in the HIV+ control group with CD4 < 100 (*N* = 15) to perform an adequate subset analysis. Ideally, future studies should be conducted comparing asymptomatic CrAg + persons to CrAg-negative persons with similar CD4 counts. CSF sampling was not performed in the asymptomatic cohort. Some asymptomatic persons would have had detectable CSF CrAg and others would have had *Cryptococcus* yeasts in the brain parenchyma in the absence of CSF CrAg detectability. However, the fundamental conclusion remains that cryptococcal antigenemia in persons with low CD4 counts, even if not a causal factor for neurocognitive impairment, can be a useful marker for identifying persons with decreased neurocognitive function. This has very important implications when enrolling these persons into HIV treatment and care as there is likely a need for increased adherence support for these persons compared to the typical HIV-infected person initiating HIV care.

The second finding of the study demonstrated that asymptomatic CrAg + persons improved on most neurocognitive measures over a period of 4 weeks. In our prior longitudinal follow-up of persons with cryptococcal meningitis, neurocognitive performance came to within one standard deviation of HIV-negative population norms (QNPZ-8 = −1.0 ± 1.1) and similar to HIV-infected controls (QNPZ-8 = −0.7 ± 1.0) after 12 months [14]. Thus, we would expect the majority of CrAg + persons to normalize their neurocognitive function over time on ART [32, 33], and any additional adherence and/or social support required is likely of short duration.

One additional contributing factor that should be considered is practice effect, especially given the one-month interval between evaluations. Practice effect has been identified in previous cohorts of HIV-infected persons undergoing neurocognitive testing [17, 34]. Comparing longitudinal changes in neurocognitive performance in both the study and the control populations would be beneficial. Unfortunately, repeat testing of neurocognitive function in our HIV-infected control population was not performed. Aside from practice effect, it is important to note that participants demonstrated improved Karnofsky performance status over the first month of ART (*P =* 0.04). The improvement in neurocognitive function is therefore potentially explained by ART initiation with improved control of HIV infection and/or the use of pre-emptive fluconazole therapy.

There are several limitations in this study that should be considered. HIV viral load was not measured in either plasma or CSF, which may act as a potential confounder for worse neurocognitive function, although plasma levels would be expected to be high [35]. In the prior cryptococcal meningitis cohort, the pre-ART plasma HIV viral load was not associated with neurocognitive dysfunction [14]. Secondly, the repeat testing of neurocognitive function which showed improvement after 1 month of ART may have a component of improvement due to a practice effect, but overall appears quite promising for longer term function. As shown previously among cryptococcal meningitis survivors, their neurocognitive function normalizes to within one standard deviation of population norms within 1 year of ART [14]. Additionally, although meningitis was excluded in the HIV-infected control population, this population was not tested for CrAg. Based on a median CD4 of 233 cells/μL, there may have been approximately 3 of 100 controls who could have tested positive, and in sensitivity analyses, this was not enough to account for the findings [8].

## Conclusions

In summary, asymptomatic CrAg + persons have worse neurocognitive function compared with their HIV-infected peers without CNS infection, although the exact etiology of poor neurocognitive function is unclear. There are a multitude of reasons for starting pre-emptive fluconazole treatment in HIV-infected CrAg + persons in the absence of meningitis. Screening is cost effective, and pre-emptive treatment with fluconazole can prevent cryptococcal meningitis and reduce early ART mortality [36]. Additional support for ART and fluconazole adherence during the first month of therapy has been associated with improved mortality [37]. As more countries roll out CrAg screening in populations with advanced HIV, some initial adherence support may be necessary due to the neurocognitive dysfunction present in CrAg + persons.

## Additional files

Additional file 1: Table S1. Neuropsychological test battery and neurocognitive domains evaluated. (PDF 330 kb)

Additional file 2: Neurocognitive outcomes in three HIV study populations. This file contains the data generated and analyzed in this publication. (CSV 107 kb)

Additional file 3: Table S2. Neurocognitive test average Z-scores for CrAg- vs. asymptomatic CrAg + vs. cryptococcal meningitis survivors. (PDF 268 kb)

## Abbreviations

ART: Antiretroviral therapy
AVL: Auditory visual learning
CES-D: Center for Epidemiologic Studies Depression
CI: Confidence interval
CNS: Central nervous system
CrAg: Cryptococcal antigen
CSF: Cerebrospinal fluid
DSB: Digit span backward
DSF: Digit span forward
GPB, D: Grooved pegboard, dominant hand
GPB, ND: Grooved pegboard, non-dominant hand
HAND: HIV-associated neurocognitive disorder
IHDS: International HIV Dementia Scale
IQR: Interquartile range
QNPZ-8: Quantitative neurocognitive performance z-score
SDM: Symbol digit modalities
UCLA: University of California, Los Angeles
WHO: World Health Organization
