# Supplementary material for: Neurocognitive function in HIV-infected persons with asymptomatic cryptococcal antigenemia: a comparison of three prospective cohorts
Source: BMC Neurol. 2017 Jun 12;17:110. doi: 10.1186/s12883-017-0878-2 (PMC5469183; doi:10.1186/s12883-017-0878-2)
Supplement: Supplementary file 1 — Neuropsychological test battery and neurocognitive domains evaluated. (PDF 330 kb) [file 12883_2017_878_MOESM1_ESM.pdf]

Additional Table 1. Neuropsychological test battery and neurocognitive domains evaluated.

Test	Test Description	Cognitive Domains
WHO-UCLA AVLT-Total*	Subjects are asked to recall a list of words. The test is similar to the Rey Auditory Verbal Learning test, however words have been selected to be recognizable to a variety of cultures	Verbal learning
Digit Span Forward and Backward	Subjects are given a series of digits of increasing length and are asked to repeat them in forward or backward order	Attention, Working memory
Semantic Verbal Fluency	Subjects are given 60 seconds to produce as many words as possible within a specific category such as 'animals'	Language fluency (Verbal)
WAIS-III Symbol Digit	Subjects are asked to match symbols to numbers as quickly as possible over 120 seconds using a visual reference.; also referred to as a Digit Symbol Substitution Test.	Speed of information processing, Concentration
Color Trails 1	Subjects connect encircled numbers scattered on a page in sequence during a set amount of time. This test is similar to the Trail Making Test but has been formulated to minimize cultural bias by not using any letters or written instructions	Speed of information processing, Attention
Color Trails 2	Similar to The Color Trails 1 but each number is printed in two different colors, and subjects are asked to maintain the numerical sequence while alternating colors	Executive function
Grooved Pegboard	Subjects are timed while placing pegs which each have a key along one side in holes in various orientations in a pegboard with either their dominant or non-dominant hand	Fine motor
Finger tapping	Subjects tap as rapidly as possible using the index finger on a specially adapted tapper for five 10-second trials	Motor speed

WHO-ULCA AVLT = World Health Organization-University of California-Los Angeles Auditory Verbal Learning test; WAIS = Wechsler Adult Intelligence Scale
