# Supplementary material for: Neurocognitive function in HIV-infected persons with asymptomatic cryptococcal antigenemia: a comparison of three prospective cohorts
Source: BMC Neurol. 2017 Jun 12;17:110. doi: 10.1186/s12883-017-0878-2 (PMC5469183; doi:10.1186/s12883-017-0878-2)
Supplement: Supplementary file 3 — Neurocognitive test average Z-scores for CrAg- vs. asymptomatic CrAg + vs. cryptococcal meningitis survivors. (PDF 268 kb) [file 12883_2017_878_MOESM3_ESM.pdf]

Additional Table 2. Neurocognitive test average Z-scores for CrAg- vs. asymptomatic CrAg+ vs. Cryptococcal meningitis survivors

Neurocognitive Test	Cohort A Cryptococcal Meningitis N=90			Cohort B Asymptomatic CrAg+ N=87			Cohort C HIV+ controls N=125			A vs. B *P*-value	B vs. C *P*-value
	N skipped	Average	SD	N skipped	Average	SD	N skipped	Average	SD		
Symbol digit modalities	24	-2.16	±1.06	20	-2.00	±1.17	0	-0.96	±1.11	0.33	<0.001
Grooved pegboard dominant	22	-1.93	±2.50	11	-0.34	±1.31	0	-0.98	±2.29	<0.001	0.02
Grooved pegboard non-dominant	22	-1.70	±2.21	11	-0.42	±1.53	0	-1.36	±3.49	<0.001	0.02
Color Trails 1	34	-3.72	±3.01	26	-2.59	±2.69	0	-2.18	±2.97	0.01	0.30
Color Trails 2	38	-4.05	±2.47	32	-3.84	±2.61	0	-2.92	±2.62	0.58	0.01
Digit span forward	2	-0.65	±1.05	1	-0.53	±0.92	0	0.17	±1.18	0.44	<0.001
Digit span backward	2	-1.55	±1.31	1	-1.63	±1.47	0	-1.15	±0.82	0.70	0.003
Auditory verbal learning	2	-2.43	±1.50	1	-2.24	±1.32	0	-1.48	±1.23	0.36	<0.001
Verbal fluency	4	-0.98	±0.63	1	-1.01	±0.73	0	-0.48	±0.63	0.78	<0.001
Finger tapping	19	-2.57	±1.50	9	-2.33	±1.16	0	-1.32	±1.12	0.24	<0.001
QNPZ-8	40	-2.22	±1.25	36	-1.80	±1.03	0	-1.36	±1.02	0.02	0.003

Numbers represent Z-scores referenced against age and education adjusted, HIV-negative Ugandan population norms. *P*-values were calculated using student's t-test. SD = standard deviation.
